# Photoimmunotherapy targeting biliary‐pancreatic cancer with humanized anti‐TROP2 antibody

**DOI:** 10.1002/cam4.2658

**Published:** 2019-11-01

**Authors:** Takashi Nishimura, Makoto Mitsunaga, Ryoichi Sawada, Masayuki Saruta, Hisataka Kobayashi, Noriko Matsumoto, Toru Kanke, Hiroyuki Yanai, Koji Nakamura

**Affiliations:** ^1^ Division of Gastroenterology and Hepatology Department of Internal Medicine The Jikei University School of Medicine Tokyo Japan; ^2^ Molecular Imaging Program Center for Cancer Research National Cancer Institute NIH Bethesda MD USA; ^3^ Drug Discovery Laboratories Chiome Bioscience Kawasaki Japan

**Keywords:** antibody‐drug conjugates, molecular‐targeted therapy, near‐infrared, photoimmunotherapy, TROP2

## Abstract

Photoimmunotherapy (PIT) is a new type of tumor‐specific treatment utilizing monoclonal antibody (mAb)‐photosensitizer conjugates and near‐infrared (NIR) light irradiation. One potential PIT target, the type I transmembrane protein TROP2, is expressed at high levels in many cancers, including pancreatic carcinoma (PC) and cholangiocarcinoma (CC), in which its expression is correlated with poor prognosis and tumor aggressiveness. In this study, we assessed the efficacy of PIT utilizing newly developed humanized anti‐TROP2 mAb conjugated to the photosensitizer IR700 (TROP2‐IR700) for PC and CC. Immunohistochemistry on PC and CC tissue microarrays confirmed that TROP2 is overexpressed in about half of PC and CC specimens. Using cultured PC and CC cells, TROP2‐IR700 localized TROP2‐specific and target‐specific cell killing was observed after NIR light irradiation. In addition, TROP2‐IR700 was localized to mouse xenograft tumors expressing TROP2 after intravenous injection. PC and CC xenograft tumor growth was significantly inhibited by TROP2‐targeted PIT relative to controls. The efficacy of TROP2‐targeted PIT in vitro and against xenografted tumors in vivo suggests promise as a therapy for human PC and CC, both of which currently have dismal prognoses and limited therapeutic options.

## Graphical abstract

The recently developed photoimmunotherapy (PIT) method targets and disrupts cancer cell membranes by combining monoclonal antibody (mAb)‐based targeting with a photosensitizer IR700. By using newly developed humanized anti‐TROP2 mAb conjugated to the IR700 (TROP2‐IR700), both pancreatic cancer and cholangiocarcinoma cells were successfully treated with near‐infrared light both in vitro and in vivo. Given that there is no molecular‐targeted therapy in the standard of care for pancreatic cancer and cholangiocarcinoma patients, TROP2‐targeted PIT is an attractive candidate for clinical trials and ultimately treatment.

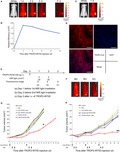

## 1 INTRODUCTION

Photoimmunotherapy (PIT) is a new class of cancer therapy based on conjugation of a monoclonal antibody (mAb) to a photosensitizing phthalocyanine dye, IRDye 700DX N‐hydroxysuccinimide (NHS) ester (IR700), followed by near‐infrared (NIR) light irradiation guided by molecular‐targeted fluorescence imaging.^1^ When exposed to NIR light, the conjugated mAb (mAb‐IR700) causes rapid and selective cell death by necrosis. A recent clinical trial of epidermal growth factor receptor (EGFR)‐targeted PIT in patients with inoperable head and neck cancer has shown promise in a Phase 1/2 clinical trial (NCT02422979).

Although the expression patterns of cancer‐specific antigens are different in each cancer type, many types of cancer are candidates for treatment using PIT. Previous studies have demonstrated that PIT is effective with a variety of different antibodies against HER2, EGFR, PD‐L1, CEA, and others.^1^, ^2^, ^3^, ^4^ Identification of additional target proteins is necessary to expand the utility of PIT for cancer treatment.

Tumor‐associated calcium signal transducer 2 (TROP2) is a 46‐kD glycoprotein initially identified in a trophoblast cancer cell line^5^ and is overexpressed in many epithelial cancers including pancreatic cancer (PC) and cholangiocarcinoma (CC).^6^, ^7^, ^8^, ^9^ It plays a multifunctional cellular role, including the transducing of cytoplasmic Ca^2+^ that depends on protein kinase C phosphorylation.^10^ Both TROP2 and the bicistronic CYCLIN D1‐Trop2 mRNA chimera have oncogenic properties.^11^, ^12^ Furthermore, the overexpression of TROP2 correlates with a poor prognosis in various cancers,^7^, ^8^ including PC,^13^ CC,^14^ gastric, colorectal, gallbladder, breast, lung, head and neck, cervical, and ovarian cancers. We have recently developed a humanized mAb raised against TROP2, which successfully treated for TROP2‐positive xenograft tumor models.

To our knowledge, there are no reports of PIT employing TROP2‐IR700, nor are there reports of employing PIT for biliary‐pancreatic cancer. Biliary‐pancreatic cancers are neoplasms with high mortality and a low rate of early diagnosis, with incidences increasing yearly.^15^, ^16^, ^17^ These tumors are one of the few cancers for which survival rates have not improved substantially over the past few decades.

At the time of diagnosis, most patients already have locally advanced or metastatic disease precluding surgical resection.^18^, ^19^ The prognosis for patients with unresectable PC and CC is poor with a median survival of less than a year,^20^ even if chemotherapy was performed. No mAbs have been used for PC and CC as anti‐cancer agents in clinical practice.

Here, we focused on TROP2, which is expressed in various cancer types, as a therapeutic target protein for PIT. The aim of this study was to investigate the expression rate of TROP2 in PC and CC and evaluate the efficacy of PIT with TROP2‐IR700 in vitro and in vivo for TROP2‐expressing PC and CC cell lines.

## 2 MATERIALS AND METHODS

### 2.1 Reagents

The HuT6‐16‐2, a humanized anti‐human TROP2 mAb (IgG1κ), was generated and provided by Chiome Bioscience, Inc (Tokyo, Japan, Patent; US9,427,464B2). Briefly, a mouse hybrodoma clone T6‐16 was identified by screening of mouse hybridoma clones raised against human TROP2. Based on the VH and VL sequences of T6‐16, generation of the humanized antibody clone, HuT6‐16‐2, was carried out according to the method of Queen et al^21^ The HuT6‐16‐2 mAb was purified by Protein A chromatography from supernatant of CHO‐DG44 cells transfected with a plasmid encoding HuT6‐16‐2. Human IgG1κ was used as an isotype control of anti‐TROP2 mAb. IR700 was obtained from LI‐COR Biosciences.

### 2.2 Immunohistochemistry

PC and CC tissue microarray (TMA) were obtained from US Biomax and Provitro, and both TROP2 and EGFR expression were determined by immunohistochemistry. TMAs were deparaffinized, and then incubated at 37°C for 5 minutes with pepsin (4 mg/mL, DAKO, Glostrup, Denmark). Endogenous peroxidase activity was inhibited by treatment with methanol containing 0.3% hydrogen peroxide (H_2_O_2_) for 20 minutes. Sections were incubated with a mouse IgG1 primary antibody against TROP2, K5‐63‐17 (10 µg/mL, Chiome Bioscience) at 4°C overnight, and then with biotinylated anti‐mouse IgG (Vector Laboratories) for 30 minutes and stained with VECTASTAIN Elite ABC kit (Vector Laboratories) according to the manufacture's protocol. Specific peroxidase activity was visualized with 3,3′‐diaminobenzidine (DAB; Nichirei Bioscience). Slides were counterstained with hematoxylin (Wako Pure Chemical Industries, Ltd.). For EGFR assay, TMAs were deparaffinized, and retrieved by autoclave treatment at 110°C for 10 minutes in citrate buffer (pH 6.0). Endogenous peroxidase activity was inhibited by treatment with methanol containing 1% H_2_O_2_ for 30 minutes. Then, sections were incubated with the primary antibody against EGFR (DAKO) diluted 1:300 in PBS at 4°C overnight. Primary antibody binding was visualized using VECTASTAIN Elite ABC kit with DAB and hematoxylin counterstain. Samples were evaluated under light microscopy. TROP2 and EGFR expressions on the plasma membranes were evaluated according to the intensity of staining; no reactivity, weak reactivity, moderate reactivity, and strong reactivity.

### 2.3 Synthesis of IR700‐conjugated antibodies

Anti‐TROP2 mAb (1.0 mg, 6.8 nmol) or human IgG1 as an isotype control (1.0 mg, 6.8 nmol) were incubated with IR700 (66.8 µg, 34.2 nmol) in 0.1 mol/L Na_2_HPO_4_ (pH 8.5) at room temperature for 1 hour. The mixture was purified with a Sephadex G50 column (PD‐10; GE Healthcare). The concentrations of protein and IR700 were determined using spectrophotometer by measuring absorption at 280 nm and 689 nm (UV‐1800; Shimadzu Corp.) to confirm the number of fluorophore molecules conjugated to each antibody molecule. The number of fluorophore molecules per antibody molecule was adjusted to approximately 3.

### 2.4 Cell culture

TROP2‐expressing human PC cell line (PK‐59 and KP‐3L) and CC cell line (TFK‐1 and HuCCT‐1) were purchased from Cell Resource Center for Biomedical Research, Institute of Development, Aging and Cancer Tohoku University. TROP2‐negative 3T3/HER2 cells were used as negative controls. Cells were cultured with RPMI 1640 (Life Technologies) supplemented with 10% fetal bovine serum and 1% penicillin/streptomycin (Life Technologies) in tissue culture flasks in a humidified incubator at 37°C in an atmosphere of 95% air and 5% carbon dioxide.

### 2.5 Comparison of antigen‐binding activity between TROP2‐IR700 and unconjugated mAb

Antigen‐binding activity of TROP2‐IR700 and unconjugated anti‐TROP2 mAb were analyzed by flow cytometry using the FACSCalibur flow cytometer (Nippon Becton Dickinson). PK‐59 cells obtained when growth was subconfluent were analyzed by flow cytometry. PK‐59 cells were incubated with TROP2‐IR700 or unconjugated mAb at several concentrations at 4°C for 20 minutes. Palivizumab (humanized anti‐respiratory syncytial virus IgG1 antibody) was used as an isotype control. The cells were washed with PBS and incubated with secondary antibody (phycoerythrin‐conjugated anti‐human IgG) at 4°C for 20 minutes followed by flow cytometry analysis. The dose‐dependent increase in antigen‐binding activity was expressed as mean fluorescence intensity (MFI) and the EC_50_ value was calculated by Microplate Manager 6 software (Bio‐Rad).

### 2.6 Fluorescence microscopy

Fluorescence microscopy was performed using an IX73 fluorescence microscope (Olympus) to confirm molecular target‐specific localization of TROP2‐IR700 in above cells. Cells were seeded on cover glass bottomed dishes and incubated for 24‐48 hours at 37°C. TROP2‐IR700 (10 µg/mL) was added to the culture medium and incubated for 3 hours at 37°C. Then, cells were washed with PBS, and fluorescence microscopy was performed with the following filter settings: 608‐648‐nm excitation filter and 672‐712‐nm emission filter for IR700.

### 2.7 Flow cytometry

To analyze the expression levels of TROP2 in above cells, IR700 fluorescence was measured by flow cytometry analysis (MACSQant analyzer; Miltenyi Biotec). Cells were seeded in 35‐mm dishes and cultured for 48 hours. After 3 hours of incubation with 10 µg/mL of TROP2 ‐IR700, the media were removed and the plates were washed with PBS. Flow cytometric analysis was subsequently performed. The MFIs were calculated and compared to the isotype control. In addition, to confirm the target specificity, unconjugated anti‐TROP2 mAb (100 µg/mL) was added to the cells to block TROP2 before incubation with the TROP2‐IR700 conjugate.

### 2.8 In vitro PIT and cytotoxicity assay

Cells were seeded on 35‐mm cell culture dishes and incubated for 48 hours at 37°C. The medium was replaced with fresh phenol red‐free RPMI 1640 containing TROP2‐IR700 (10 µg/mL). Cells were incubated for another 24 hours at 37°C, washed with PBS, and added fresh phenol red‐free RPMI 1640. Cells were irradiated with NIR light using a 690‐nm continuous wave laser (ML6540‐690; Modulight, Inc). Power density of 29.5 mW/cm^2^ was measured with an optical power meter (PM 100; Thorlabs). The doses of irradiation for each dish were 0, 1, 2, 4, 8, and 16 J/cm^2^, respectively. After irradiation, cells were collected and resuspended with PBS, followed by LIVE/DEAD® cytotoxicity assay (Life Technologies) which can detect damaged cellular membranes.

### 2.9 Xenograft tumor model

All animal studies were conducted in accordance with the guidelines established by the Animal Care Committee of the Jikei University School of Medicine and Chiome Bioscience. Six‐week‐old female BALB/cAJcl‐nu/nu nude mice (Clea Japan Inc) were allowed to acclimatize and recover from shipping‐related stress for 1 week before the studies, and were kept under a controlled light‐dark cycle (12:12 hours). Five million of PK‐59 or TFK‐1 cells were injected subcutaneously into the right dorsum of the mice. Five million of 3T3/HER2 cells were injected subcutaneously into the left dorsum of the mice as negative control. The tumor xenografts were measured with an external caliper, and the tumor volume was calculated using the following formula: length × width ×height × 0.5.^22^

### 2.10 In vivo fluorescence imaging

To determine the biodistribution of TROP2‐IR700, fluorescence images were obtained with the IVIS® Imaging System (Caliper Life Sciences) using a 675‐nm excitation filter and a 695‐770‐nm emission filter. Xenograft tumors reached approximately 100 mm^3^ after subcutaneous injection of 5 × 10^6^ PK‐59 cells was used in this study. Fluorescence images were obtained serially starting 1 day after injection of 200‐µg TROP2‐IR700 under the same setting (eg, exposure time, camera binning, and stage height), and fluorescence intensities in the target tumor were analyzed. All fluorescence images were analyzed with Living Image® Software. The region interest was manually determined on each tumor area depending on where the IR700 fluorescence was localized.

### 2.11 Microdistribution analysis of fluorescence probe‐labeled antibody in PK‐59 tumor xenografts

To investigate the tumor microdistribution of anti‐TROP2 mAb in harvested PK‐59 tumors, 200‐µg TROP2‐Cy5 were injected intravenously into PK‐59 tumor‐bearing mice. PK‐59 tumors were harvested and fixed overnight in 4% paraformaldehyde dissolved in 0.01 mol/L PBS, immersed in graded concentrations of sucrose in PBS (10% for 1 hour, 20% for 1 hour), embedded in OCT compound, and stored at −80°C. Microscopic fluorescence imaging of frozen tissue sample sections were acquired as 10‐µm slice sections on glass slides with nuclear counter staining by 4′,6′‐diamidino‐2‐phenylindole (DAPI). Confocal imaging was performed with a ZEISS LSM 880 (Carl Zeiss). To detect DAPI and Cy5 fluorescence, 405 and 633 nm lasers were used for excitation, and emitted fluorescence was acquired with band pass filter settings of 410‐498 and 637‐755 nm, respectively.

### 2.12 In vivo PIT

In vivo experiments were conducted with mice transplanted with PK‐59 and TFK‐1 cells respectively. Mice‐ bearing tumor xenograft reaching approximately 100 mm^3^ in volume after subcutaneous injection of 5 × 10^6^ PK‐59 or TFK‐1 cells. PK‐59 tumor‐bearing mice were randomized into following six groups (n = 10 mice in each group): (a) no treatment (iv injection of PBS without NIR light irradiation); (b) iv injection of PBS followed by NIR light irradiation (30 J/cm^2^) 1 and 2 days after injection; (c) iv injection of 200‐µg unconjugated anti‐TROP2 mAb without NIR light irradiation; (d) iv injection of 200‐µg unconjugated anti‐TROP2 mAb followed by NIR light irradiation (30 J/cm^2^) 1 and 2 days after injection; (e) iv injection of 200‐µg TROP2‐IR700 without NIR light irradiation; (f) iv injection of 200‐µg TROP2‐IR700 followed by NIR light irradiation (30 J/cm^2^) 1 and 2 days after injection. These therapies were performed every week for up to 2 weeks. TFK‐1 tumor‐bearing mice were randomized into following five groups (n = 10 mice in each group): (a) no treatment (iv injection of PBS without NIR light irradiation); (b) iv injection of PBS followed by NIR light irradiation (30 J/cm^2^ on day 1 and 50 J/cm^2^ on day 2); (c) iv injection of 200‐µg unconjugated anti‐TROP2 mAb without NIR light irradiation; (d) iv injection of 200‐µg isotype control IgG conjugated to IR700 followed by NIR light irradiation (30 J/cm^2^ on day 1 and 50 J/cm^2^ on day 2); (e) iv injection of 200‐µg TROP2‐IR700 followed by NIR light irradiation (30 J/cm^2^ on day 1 and 50 J/cm^2^ on day 2). These treatments were performed every week for up to 3 weeks. NIR light irradiation was performed under isoflurane anesthesia with a 690‐nm continuous wave laser at a power density of 330 mW/cm^2^. After the treatments, tumor volumes were measured two or three times a week until the volume reached 1000 mm^3^.

### 2.13 Statistical analyses

Data are expressed as means ± SEM from a minimum of three experiments. Statistical analyses were carried out using GraphPad Prism software (GraphPad Software Inc). Student's *t* test was used to compare the two treatment groups. For multiple comparisons, one‐way analysis of variance (ANOVA) followed by Dunnett's test was used for comparison to control group. The correlation between TROP2 and EGFR was analyzed using Spearman's rank correlation coefficient. A value of *P* < .05 was considered statistically significant.

## 3 RESULTS

### 3.1 TROP2 is overexpressed in PC and CC, but its expression is not correlated with EGFR expression

**Figure 1:**
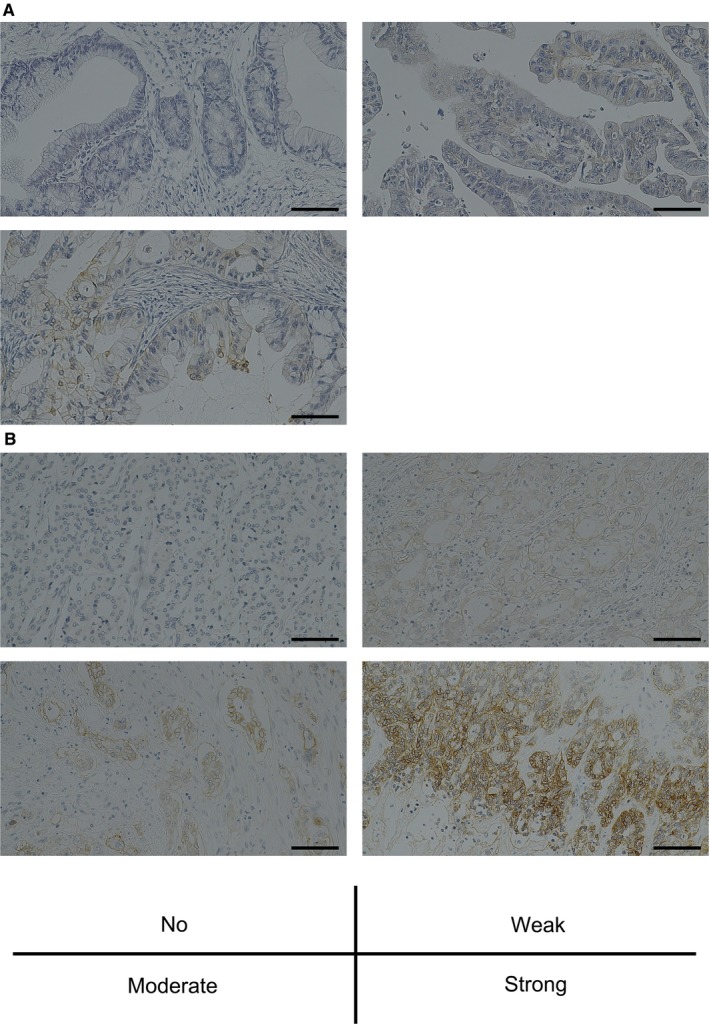
Representative immunohistochemical staining (IHC) for TROP2. A, IHC for pancreatic cancer (PC) specimens: *Upper left*; absent TROP2 expression. *Upper right*; weak TROP2 expression. *Lower left*; moderate TROP2 expression. B, IHC for cholangiocarcinoma (CC) specimens: *Upper left*; absent TROP2 expression. *Upper right*; weak TROP2 expression. *Lower left*; moderate TROP2 expression. *Lower right*; strong TROP2 expression. Scale bar: 200 μm

Immunohistochemical staining of tissue microarrays was used to evaluate TROP2 expression in PC and CC tumor samples. Representative staining for TROP2 is shown in Figure 1. Moderate or strong membranous expression of TROP2 was detected in 40% of PC specimens and 46% of CC specimens. In PC specimens, strong TROP2 expression was observed in none, moderate expression in 40%, weak expression in 40%, and no detectable expression in 20% of the specimens. In CC specimens, strong TROP2 expression was observed in 9%, moderate expression in 37%, weak expression in 31%, and no expression in 22% of cases (Table 1). For PC, strong EGFR expression was observed in 2.5%, moderate expression in 5%, weak expression in 32.5%, and no expression in 47.5% of specimens (Table 2). In contrast, for CC strong EGFR expression was observed in 13%, moderate expression in 33.3%, weak expression in 7.4%, and no detectable expression in 27.8% of specimens (Table 3). There were no significant correlations between the expression of TROP2 and EGFR in either PC or CC tumors. In 10 normal pancreatic tissue specimens, strong TROP2 expression was not observed, whereas moderate expression was observed in 20% of specimens, weak expression in 20%, and no detectable expression in 60%. Collectively, TROP2 was expressed at a higher level in PC than in normal pancreas tissues. Additionally, TROP2 expression was not observed in PC tissue stroma.

**Table 1:** Immunohistochemical staining of tissue microarrays of pancreatic cancer and cholangiocarcinoma to evaluate TROP2 expression

	No. of patients	TROP2 expression (%)			
		No	Weak	Moderate	Strong
Pancreatic cancer	40	8 (20)	16 (40)	16 (40)	0 (0)
Cholangiocarcinoma	54	12 (22)	17 (31)	20 (37)	5 (9)

**Table 2:** Correlation between expression of TROP2 and EGFR in pancreatic cancer tissues

EGFR	TROP2				Total	r	*P*‐value
	0	1+	2+	3+			
0	2	8	9	0	19	−0.11	.52
1+	4	7	2	0	13		
2+	2	0	5	0	2		
3+	0	1	0	0	1		
Total	8	16	16	0	40		

**Table 3:** Correlation between the expression of TROP2 and EGFR in cholangiocarcinoma tissues

EGFR	TROP2				Total	r	*P*‐value
	0	1+	2+	3+			
0	1	6	7	1	15	−0.19	.17
1+	4	3	6	1	4		
2+	4	5	6	3	18		
3+	3	3	1	0	7		
Total	12	17	20	5	54		

### 3.2 Binding activity of TROP2‐IR700 is reduced compared to unconjugated mAb

The binding affinity of TROP2‐IR700 and unconjugated mAb to TROP2 antigen expressed on PK‐59 cells were measured and compared by flow cytometry analysis. As shown in Figure S1, the binding activity of TROP2‐IR700 was reduced compared to unconjugated mAb. The EC_50_ value of TROP2‐IR700 was 6.38 × 10^−8^ mol/L, which was higher than that of unconjugated mAb (1.20 × 10^−8^ mol/L).

### 3.3 TROP2 expression in human PC and CC cells in vitro

**Figure 2:**
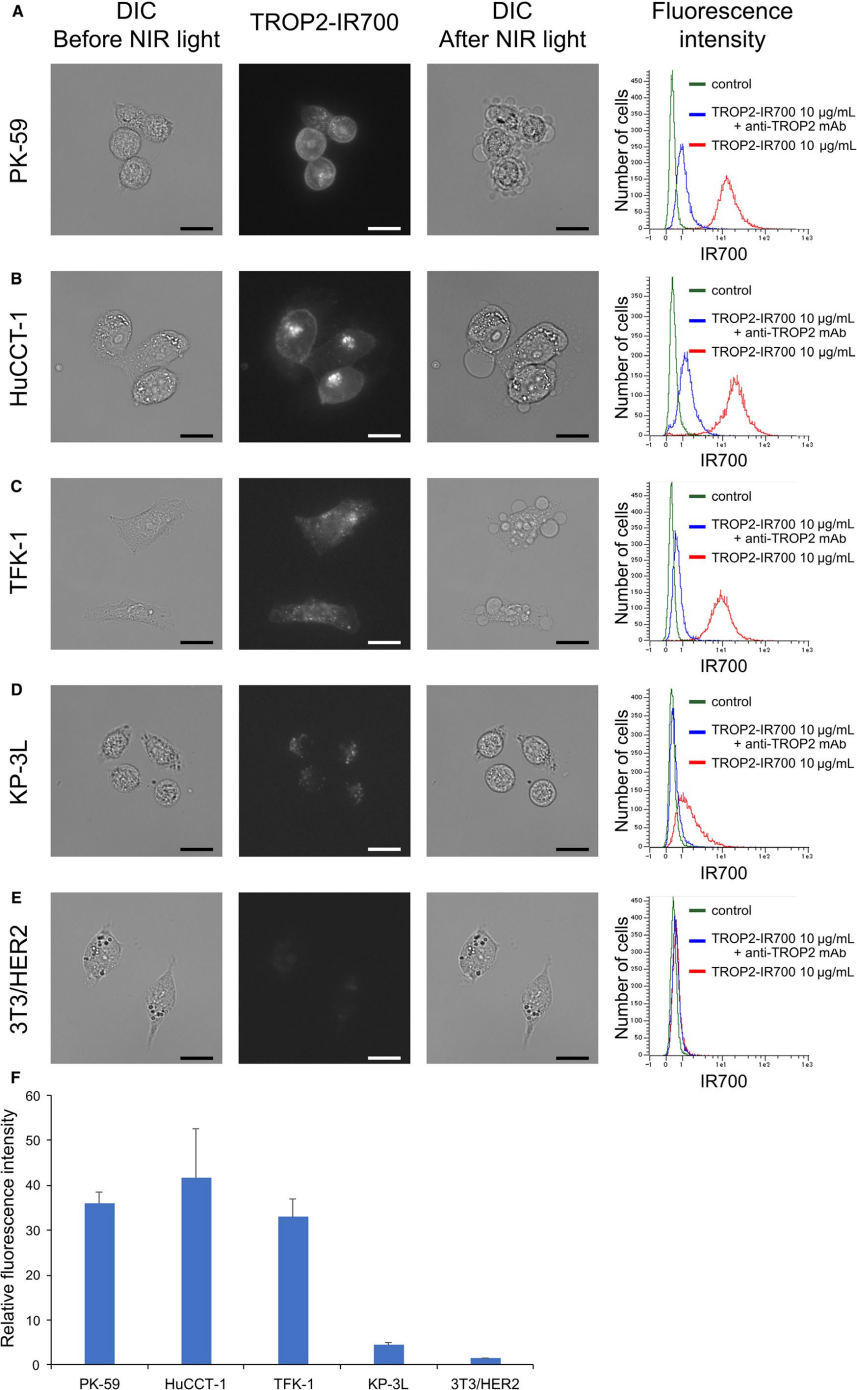
Localization and expression of TROP2 in PC and CC cells in vitro. Strong localization and TROP2‐specific binding of TROP2‐IR700 was observed in PK‐59 (A), HuCCT‐1 (B) and TFK‐1 (C) cells by fluorescence microscopy and flow cytometry analysis, and slight localization and specific binding of TROP2‐IR700 in KP‐3L (D), These fluorescence signals were colocalized on cell surface and cytosol. TROP2‐specificity was demonstrated by adding of excess unconjugated antibody. On the other hand, TROP2‐negative 3T3/HER2 cells (E) showed neither localization nor specific binding of TROP2‐IR700. DIC: differential interference contrast. Scale bar: 20 μm. F, Relative fluorescence intensity of TROP2‐IR700 in PK‐59, HuCCT‐1, TFK‐1, KP‐3L and 3T3/HER2 cells. Data are presented as the means ± SEM (n = 3)

Binding of TROP2‐IR700 to human PC and CC cells was analyzed by fluorescence microscopy and flow cytometry. PK‐59, HuCCT‐1, and TFK‐1 cells showed strong cytosolic and cell‐surface localization of TROP2‐IR700 (Figure 2A‐C), but KP‐3L cells had lower signals (Figure 2D). These signals were blocked by adding excess unconjugated anti‐TROP2 mAb (Figure 2A‐D). In contrast, TROP2‐IR700 fluorescence was not detected in TROP2‐negative 3T3/HER2 cells (Figure 2E). The ratios of the MFI relative to the isotypic control were 35 ± 2.5 in PK‐59 cells, 41 ± 11 in HuCCT‐1 cells, 33 ± 4.1 in TFK‐1 cells, 4.6 ± 0.4 in KP‐3L cells, and 1.4 ± 0.1 in 3T3/HER2 cells, respectively (means ± SEM, n = 3) (Figure 2F).

### 3.4 TROP2‐targeted PIT for PC and CC in vitro

**Figure 3:**
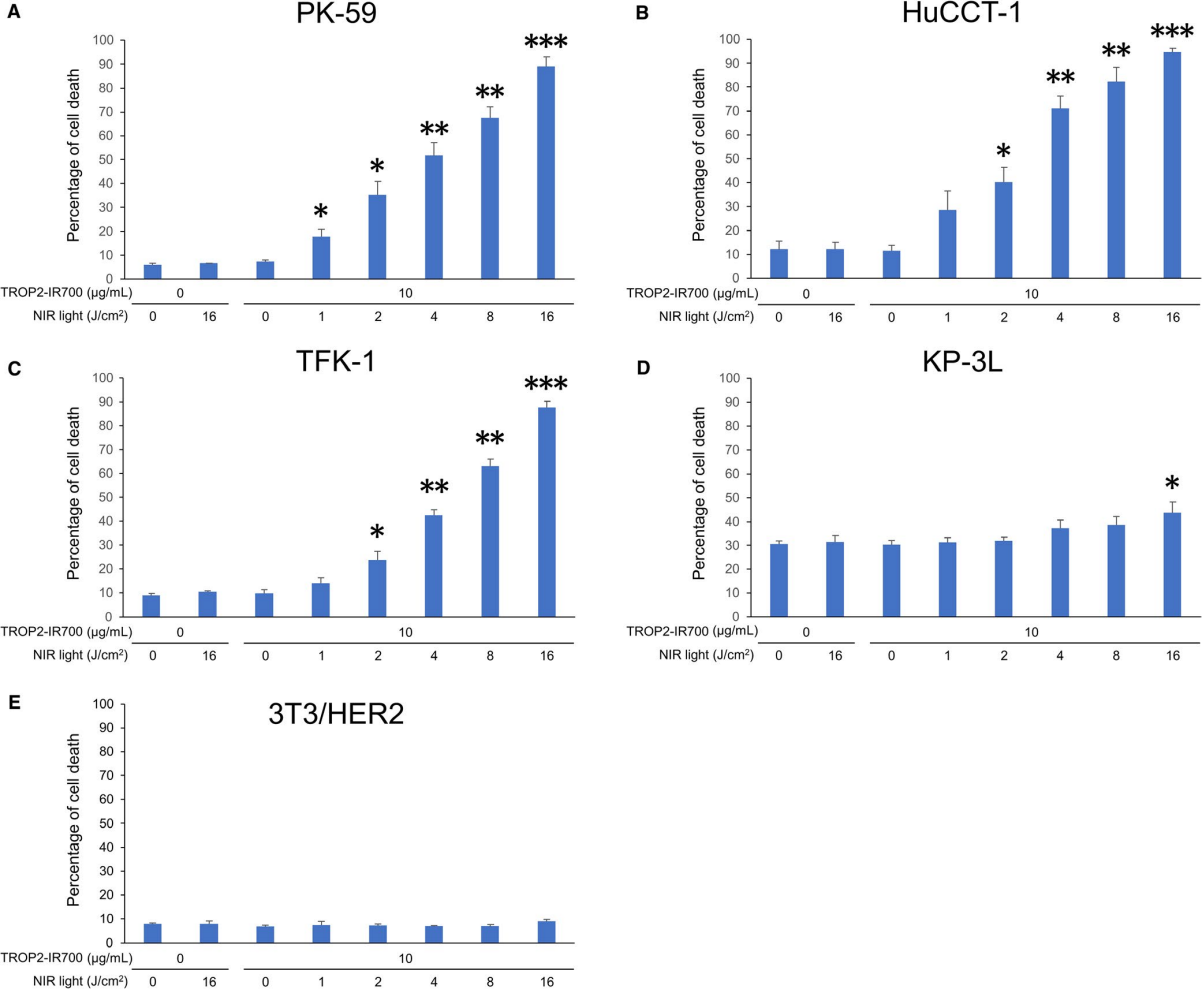
Evaluation of cell damage by PIT in vitro with anti‐TROP2 mAb‐IR700. Induction of near‐infrared (NIR) light dose‐dependent cell death in PK‐59 (A), HuCCT‐1 (B), TFK‐1 (C), and KP‐3L (D) cells by photoimmunotherapy (PIT) with TROP2‐IR700 as shown by the percentage of cell death. Data are presented as the means ± SEM (n = 3, *P < .05, **P < .005, ***P < .0001, vs untreated control, Student's *t* test). E, TROP2‐negative 3T3/HER2 cells indicates lack of cytotoxicity associated with TROP2‐IR700 treatment or TROP2‐targeted PIT

Previous studies showed that tumor cell death in response to PIT is rapidly induced through membrane damage, which can be detected by the LIVE/DEAD assay.^1^ The rate of cell death was increased in a NIR light dose‐dependent manner in TROP2‐positive PK‐59, HuCCT‐1, and TFK‐1 cells (Figure 3A‐C). Moreover, cells exhibited bleb formation immediately after NIR irradiation (Figure 2A‐C). On the other hand, in KP‐3L cells cell death was only slightly increased even when the NIR light dose was increased to 16 J/cm^2^ (Figure 3D) and cells did not form blebs (Figure 2D). Cell death and morphological changes were not induced by PIT in control TROP2‐negative 3T3/HER2 cells (Figures 2E and 3E). There was no significant cytotoxicity associated with NIR light alone in the absence of TROP2‐IR700 nor with TROP2‐IR700 without NIR light. We also analyzed the production of reactive oxygen species (ROS) in PK‐59 cells treated with TROP2‐IR700 followed by NIR light using CellROX® Green Reagent (Life Technologies) by fluorescence microscopy. While there were no significant changes in ROS production following incubation with TROP2‐IR700 for 3 hours without NIR light compared to untreated control, increases in ROS production were observed in the nuclei of the cells treated with TROP2‐IR700‐mediated PIT (Figure S2).

### 3.5 In vivo distribution of TROP2‐IR700

**Figure 4:**
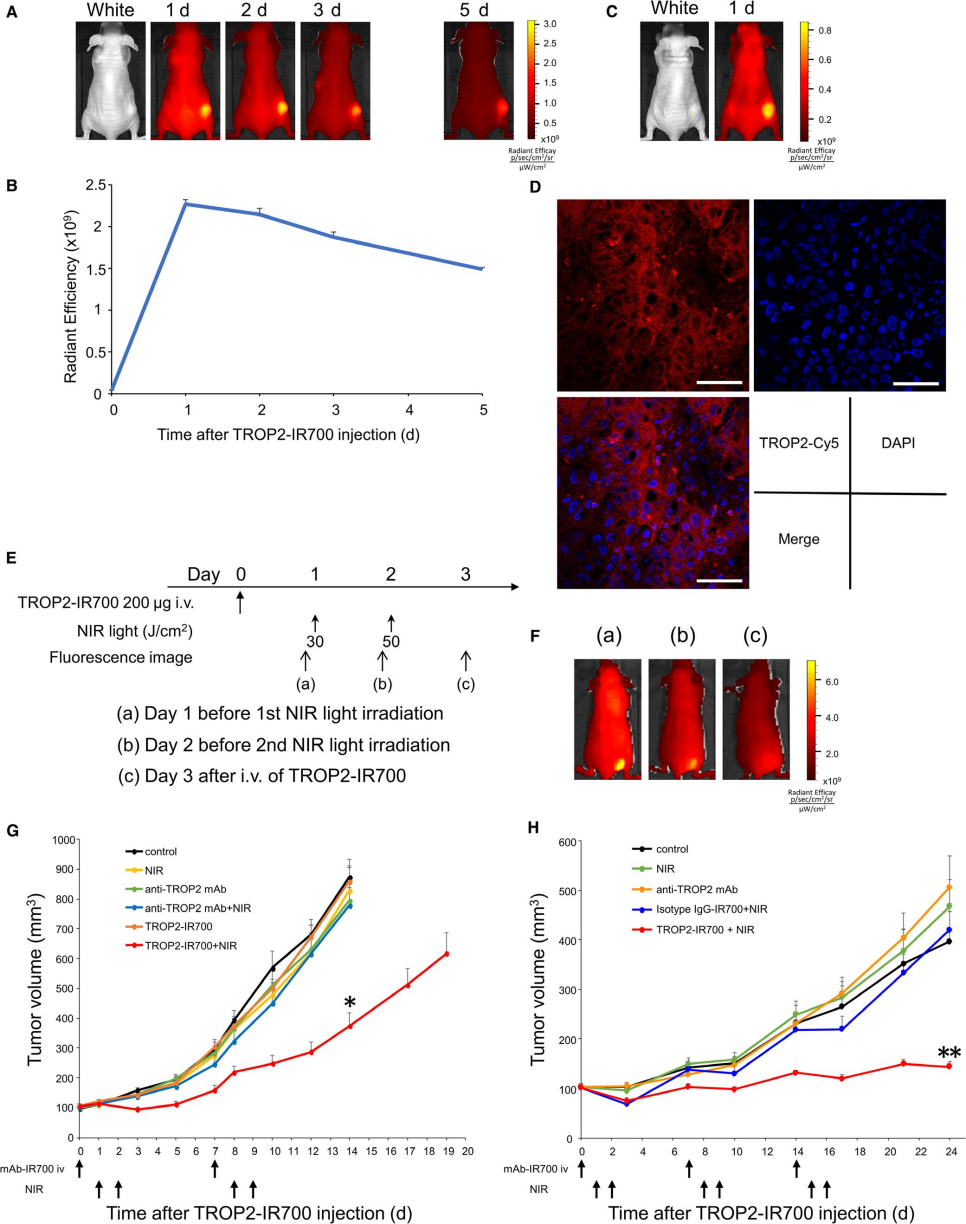
In vivo biodistribution of TROP2‐IR700 and phototherapeutic effect of PIT. A, PK‐59 tumor xenografts visualized with IR700 fluorescence after intravenous injection of 200‐µg TROP2‐IR700. B, Quantitative analysis of IR700 fluorescence signal radiant efficiency detected in PK‐59 tumors following injection of TROP2‐IR700 injection over time. Data are presented as the means ± SEM (n = 3). C, PK‐59 tumors (right dorsum) were selectively visualized with IR700 fluorescence after intravenous injection of TROP2‐IR700, whereas TROP2‐negative 3T3/HER2 tumors (left dorsum) did not show apparent fluorescence signals. D, Microdistribution of anti‐TROP2 antibody in PK‐59 tumors was shown. TROP2‐Cy5 fluorescence was detected on the PK‐59 tumor cell surface and cytosol. Scale bar = 50 µm. E and F, The treatment regimen were shown. Images were obtained at indicated time point. Representative images of TFK‐1 tumor‐bearing mice before and after NIR light irradiation were shown. G, Tumor volume of PK‐59 in the groups of TROP2‐IR700 with NIR light was significantly reduced compared to the untreated control group. Data are presented as the means ± SEM (n = 10 in each group, 14 days after initial treatment; *P < .0001: TROP2‐IR700 with NIR light vs untreated control; Dunnett's test with ANOVA). H, Tumor volume of TFK‐1 in the groups of TROP2‐IR700 with NIR light was significantly reduced compared to the untreated control group. Data are presented as the means ± SEM (n = 10 in each group, 24 days after initial treatment; **P < .005: TROP2‐IR700 with NIR light vs untreated control; Dunnett's test with ANOVA)

To further confirm the target‐specific localization of TROP2‐IR700 in vivo, serial fluorescence images were obtained 1, 2, 3, and 5 days after intravenous injection of TROP2‐IR700 in PK‐59 tumor xenograft models by in vivo fluorescence imaging system. The PK‐59 tumors were specifically visualized with IR700 fluorescence (Figure 4A). Serial image analysis showed that maximum IR700 signals were obtained 1 day after mAb‐IR700 injection, and the signal decreased gradually over the following days (Figure 4B). Next, to examine target specificity, TROP2‐IR700 was injected to mice bearing both PK‐59 and TROP2‐negative 3T3/HER2 tumors. TROP2‐IR700 selectively localized in PK‐59 tumors but not in 3T3/HER2 tumors (Figure 4C).

### 3.6 Tumor microdistribution of TROP2 in PK‐59 tumors

To investigate anti‐TROP2 mAb tumor microdistribution in PK‐59 xenografts, tumors were excised 1 day after injection of Cy5‐conjugated anti‐TROP2 mAb (TROP2‐Cy5), then confocal images of frozen tumor sections were acquired. TROP2‐Cy5 was detected mainly on the tumor cell surface and in cytosol (Figure 4D).

### 3.7 TROP2‐targeted PIT for human PC and CC cells in vivo

The treatment regimen and imaging protocol are shown in Figure 4E. Images were taken three times after intravenous injection of TROP2‐IR700 in TFK‐1 tumor xenograft tumor models. First images were obtained 1 day after TROP2‐IR700 injection just before NIR light irradiation, second images were obtained 2 days after TROP2‐IR700 injection just before second NIR light irradiation, and third image was obtained 3 days after TROP2‐IR700 injection. Tumor‐selective TROP2‐IR700 fluorescence signals were obtained on day 1 and day 2 just before NIR light irradiation (Figure 4F).

The presence of a therapeutic effect in response to TROP2‐IR700‐mediated PIT was assessed in PK‐59 or TFK‐1 tumor‐bearing mice with multiple controls. Mice with xenografts of approximately 100‐mm^3^ volume were randomized into six and five groups, respectively, then intravenously injected with TROP2‐IR700. Tumors were irradiated with NIR light on 1 and 2 days postinjection. Significant reductions in the volumes of PK‐59 tumors were observed in the experimental group relative to the negative control group (n = 10 in each group, 14 days after initial treatment; *P < .0001; TROP2‐IR700 with NIR light vs untreated control; Dunnett's test with ANOVA). Likewise, the volumes of TFK‐1 tumors were significantly reduced by TROP2‐IR700 treatment followed by NIR light irradiation (n = 10 in each group, 24 days after initial treatment; **P < .005: TROP2‐IR700 with NIR light vs untreated control; Dunnett's test with ANOVA) (Figure 4G,H). Tumor volumes were controllable by repeating PIT in TFK‐1 tumors without significant side effects. Furthermore, pathological analysis revealed few viable PK‐59 cells after TROP2‐IR700‐mediated PIT, and massive granulation with inflammatory changes was observed in the tumor nodules (Figure S3).

## 4 DISCUSSION

Previous studies on TROP2 expression in PC and CC have demonstrated that the overexpression of TROP2 was detected in 55% of 197 PC specimens and 61% of 70 CC specimens.^13^, ^14^ In this study, high expression of TROP2 was detectable in 40% of PC samples and 46% of CC samples, fairly consistent with previous studies. This implies that TROP2‐targeted PIT might be considered for approximately half of PC and CC patients. Furthermore, we showed that there was no significant correlation between the expression of TROP2 and EGFR in PC and CC samples. As previous studies demonstrated the efficacy of combination PIT,^23^, ^24^ the lack of correlation suggests that a combination PIT utilizing both TROP2‐IR700 and anti‐EGFR mAb‐IR700 could significantly increase treatment efficacy. Moreover, in vivo PIT targeting stroma cells in the tumor have also been developed.^25^, ^26^ In particular, PC is recognized as a tumor with high stromal content. Therefore, a combination PIT targeting the stroma and cancer cells would be an effective strategy for treating PC.

TROP2 has been identified as a target antigen for antibody‐drug conjugates (ADC) including sacituzumab govitecan. This ADC has the humanized anti‐TROP2 mAb conjugated to a toxic payload, SN‐38 (7‐ethyl‐10‐hydroxycamptothecin, the active metabolite of irinitecan). SN‐38 is a type I topoisomerase inhibitor that causes double‐stranded DNA breaks and apoptosis, which has been reported to be active in patients with advanced, metastatic triple‐negative breast cancer (mTNBC) and metastatic nonsmall‐cell lung cancer (mNSCLC).^27^, ^28^ In general, ADC treatment has undesired side effects caused by the expression of the target antigen on normal tissue. In those trials, sacituzumab govitecan was well tolerated as well as in animal models,^29^ and it was suggested that TROP2 may be poorly accessible in normal tissues. This is related to the fact that tumors tend to have immature, leaky vessels that allow the passage of macromolecules more easily than do vessels of normal tissues.^30^ TROP2 was also expressed in normal tissues of the pancreas at a lower rate than tumors. Therefore the normal tissue toxicity would be limited when performing TROP2‐IR700‐mediated PIT for PC. As with the ADC treatment, PIT employing TROP2‐IR700 for PC and CC was expected to be an attractive, cancer‐specific treatment.

Next, we performed in vitro and in vivo experiments using human PC and CC cancer cell lines to determine the efficacy of TROP2‐IR700‐mediated PIT. Cells with strong expression of TROP2 such as PK‐59, TFK‐1, and HuCCT‐1 were photodamaged by TROP2‐IR700 with NIR light and formed blebs. In contrast, KP‐3L cells with weak expression of TROP2 were only partly photodamaged even when strong NIR light of 16 J/cm^2^ and did not form blebs. Control TROP2‐negative 3T3/HER2 did not sustain photodamage. These results were consistent with previous studies showing a correlation between the magnitude of binding of mAb‐IR700 to the target cells with a stronger PIT effect.^31^ One of the major mechanisms of PIT is considered to be a photochemical reaction that causes a change in the shape of antibody‐antigen conjugates after NIR light irradiation, leading to physical stress within the cellular membrane. This stress results in cell bursting and necrotic cell death due to increase in transmembrane water flow.^32^ Increased ROS activity was found in the nucleus of cells treated with TROP2‐IR700‐mediated PIT, consistent to that observed in cells treated with photodynamic therapy (PDT).^33^ However, this results may not be a central role of PIT phototoxicity, as the mode of cell death in response to PIT was not apoptotic but necrotic.

Moreover, we confirmed that TROP2‐Cy5 fluorescence was detected on the PK‐59 tumor cell surface and cytosol of the harvested tumors from TROP2‐Cy5‐injected mice. Then, the anti‐tumor growth reduction and survival improvement were evidenced by cell membrane damage through an increased amount of activated IR700,^31^ in contrast, control isotype IgG‐IR700 did not induce significant treatment effect upon NIR light irradiation. Consistent with previous studies, repeated PIT with TROP2‐IR700 enhanced treatment effects without adverse effects in vivo.^34^ As TROP2 is overexpressed in many types of cancer, PIT utilizing TROP2‐IR700 solely or in combination with other mAb‐IR700 could be an effective platform for multiple tumor types.

One negative aspect of PIT is that it depends on both access of the mAb‐IR700 conjugate to the target tissue and the ability to deliver NIR light. NIR light can be easily delivered to the body surface, but it is much more difficult to deliver NIR light to internal tumors such as PC and CC. To address this issue, we are considering employing fiber diffusers that can deliver NIR light deep into tissues of the body, using endoscopes, catheters, or needles for subcutaneous insertion. PDT has been conducted and established as a technically feasible method for the treatment of PC and CC patients.^35^, ^36^, ^37^ NIR light could be delivered to PC and CC patients in the case of PIT by the same routes. The primary limitation of PDT is lack of tumor specificity leading to unwanted skin photosensitivity; PIT has been developed to overcome this issue as a cancer‐specific molecular‐targeted therapy with the potential to target cells selectively without damaging surrounding normal tissues.

In conclusion, we have demonstrated that PIT utilizing TROP2‐IR700 is effective for TROP2‐expressing human PC and CC cell lines in vitro and in vivo. Given that there is no molecular‐targeted therapy in the standard of care for PC and CC patients and that TROP2 is overexpressed in about half of PC and CC specimens, TROP2‐targeted PIT is an attractive candidate for clinical trials and ultimately treatment.

## CONFLICT OF INTEREST

NM, TK, HY, and KN are employees of Chiome Bioscience.

## AUTHORS' CONTRIBUTIONS

MM and TK were involved in conceptualization. TN, MM, RS, NM, TK, HY and KN were involved in data curation. TN, MM, NM, and TK were involved in formal analysis. HK and MS were involved in supervision. TN, MM, HK, and TK were involved in writing.

## ETHICS APPROVAL AND CONSENT TO PARTICIPATE

This study was approved by The Jikei University School of Medicine.

## CONSENT FOR PUBLICATION

Not applicable.

## Data Availability

Literature collection was performed using PubMed. Statistical analyses were executed by GraphPad Prism software. Raw and processed data are stored in corresponding author and are available upon request.
