# Supplementary material for: Photoimmunotherapy targeting biliary‐pancreatic cancer with humanized anti‐TROP2 antibody
Source: Cancer Med. 2019 Nov 1;8(18):7781–92. doi: 10.1002/cam4.2658 (PMC6912056; doi:10.1002/cam4.2658)
Supplement: Supplementary file 1 [file CAM4-8-7781-s001.docx]

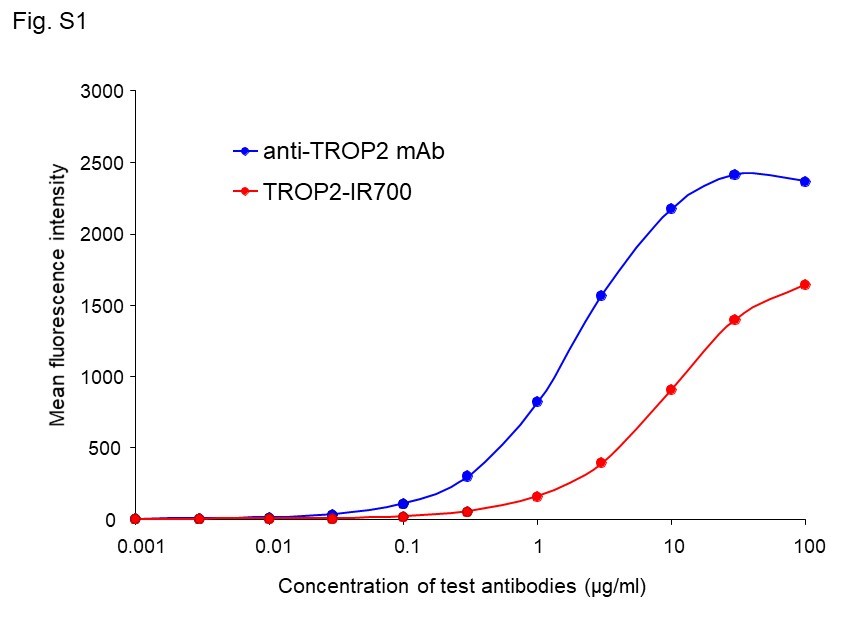


**Supplementary Figure 1.** Antigen binding activity of TROP2-IR700 and unconjugated anti-TROP2 mAb. Binding activity of TROP2-IR700 was reduced compared to unconjugated mAb.


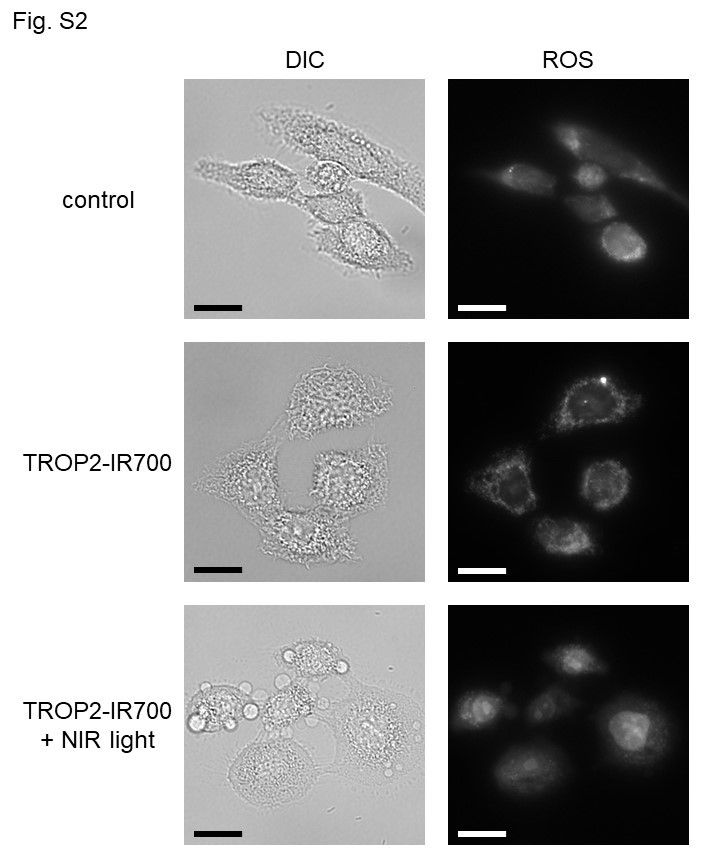


**Supplementary Figure 2.** ROS production analysis in response to PIT. ROS assay was performed to detect the intracellular ROS production using fluorogenic probe designed to measure ROS by fluorescence microscopy. While there were no significant changes in ROS production following incubation with TROP2-IR700 for 3 h without NIR-light compared to untreated control, increases in ROS production were observed in the nuclei of the cells treated with TROP2-IR700-mediated PIT. Scale bar: 20 μm.


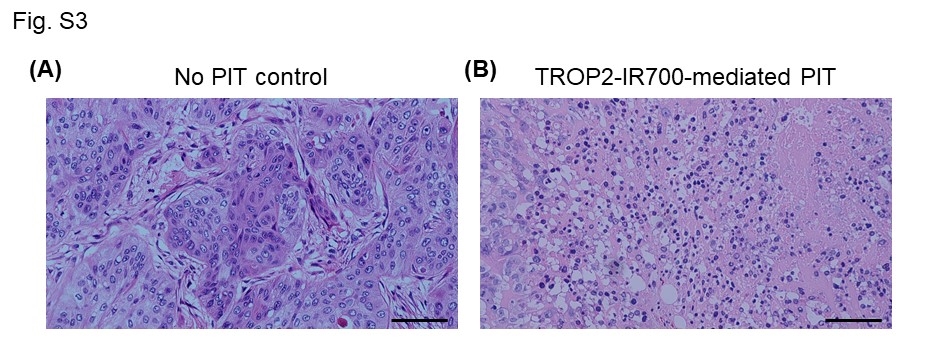


**Supplementary Figure 3.** Histological analysis of the PIT-treated tumor. A, untreated control tumor. B, TROP2-IR700-mediated PIT tumor, showing that induced large areas of necrotic cell death and granulation. Scale bar: 100 μm.
